# Evaluation of the Prevalence and Correlated Factors for Decreased Bone Mass Density among Pre- and Post-menopausal Educated Working Women in Saudi Arabia

**Published:** 2014-09

**Authors:** Samira M. Mahboub, May N. Al-Muammar, Azza A. Elareefy

**Affiliations:** ^1^Department of Tropical Health, High Institute of Public Health, Alexandria University, Egypt; ^2^Community Health Sciences Department, Applied Medical Sciences College, King Saud University, Kingdom of Saudi Arabia; ^3^Nutrition and Food Science Department, College of Home Economics, Helwan University, Egypt

**Keywords:** Calcium, Fertility, Gynaecological age, Osteoporosis, Parity, Pre-menopausal, Soft drink, Saudi Arabia

## Abstract

Most of the previous studies on osteoporosis have focused on post-menopausal women, and more research is needed to evaluate its prevalence in pre-menopausal women. This study was carried out to evaluate the prevalence and correlated factors for decreased bone mass density among pre- and post-menopausal women. This was a cross-sectional study carried out in Applied Medical Sciences College under King Saud University. All pre- and post-menopausal women working there were invited to participate in the study. Measurement of bone mass density was done by quantitative ultrasound densitometry. One-fourth of the pre-menopausal females had osteopaenia. There was a significant correlation between having osteoporosis and increasing age, fertility period, parity, menopausal duration, gynaecological age, and presence of co-morbidity, especially hypertension and diabetes mellitus. Pre-menopausal females had high prevalence of osteopaenia (24.8%), and it is recommended to implement health education campaigns demonstrating the preventive measures of osteoporosis.

## INTRODUCTION

Osteoporosis represents a major public health concern worldwide. In the United States, an estimated 10 million adults aged 50 years or more had osteoporosis, with more than 5 million having osteoporosis of the femoral neck, including 4.5 million women and 800,000 men (1). Among Arab countries, high prevalence of osteopaenia and osteoporosis were reported in Kuwaiti women aged 50 years or more (26.8% and 9.9% respectively) (2) while even higher figures were reported in the Kingdom of Saudi Arabia (KSA) among women of the same age-group, where 31% had osteopaenia, and 40% had osteoporosis at the lumbar spine (3). These high rates in KSA indicate the importance of studying the underlying risk factors in order to plan for preventive measures.

Osteoporosis is a skeletal disorder characterized by compromised bone strength predisposing to an increased risk of fracture (4). Osteoporosis is characterized by a decrease in bone mass density (BMD), and this decrease leads to structural deterioration and loss of connectivity between bone tissue, resulting in enhanced fragility and an increased risk of fracture (5).

Beginning with menopause, women sustain an accelerated period of bone loss because approximately 5-7 years after menopause, they may lose bone at the rate of 3-5% per year. It has been estimated that approximately 75% of bone lost in the years after menopause may be related to estrogen deficiency rather than age (6). Low education and being housewife were shown to be risk factors of osteoporosis. The reason probably is the effect of education on lifestyle, nutrition and economic status (7). Most of the previous studies on osteoporosis have focused on post-menopausal females, and more research is needed to evaluate its prevalence and related factors in pre-menopausal females. So, the aim of this study was to evaluate the prevalence and correlated factors of decreased bone mass density among pre- and post-menopausal educated working women in order to clarify areas for intervention at early stage for primordial and primary prevention of osteoporosis at old age.

## MATERIALS AND METHODS

### Study area and design

This was a cross-sectional study carried out in the College of Applied Medical Sciences (CAMS) (female section in Oleisha), King Saud University, Riyadh, KSA, during May to June 2012.

### Study population

All females (employees and staff members) in the college were asked to participate in the study (256 females, after exclusion of those in scholarship); 153 of them agreed to participate and gave their consent.

### Data collection

Data collection was done using a self-administered questionnaire entailing data about sociodemographic state, sun exposure, exercise, and fertility history, such as parity (number of sons and daughters), fertility period (time since menarche for pre-menopause and time since menarche to menopause for post-menopausal women), gynaecological age (time since menarche in years), and menopausal duration (time since menopause in years). Dietary assessment was done using a semi-quantitative food frequency questionnaire containing calcium-rich food items (each food item was presented in a specified amount, e.g. one egg, 1 cup of milk—300 mL, etc.), and frequency of its consumption was suggested, i.e. once daily, 3-5 times weekly, 1-3 times weekly, 1-3 times monthly, and none. Weighting of consumption frequency of each food item was done according to Willett W; each response was coded to reflect its corresponding daily consumption, e.g. once daily was coded 1 (corresponding to 1/day), 3-5 times weekly would mean, on average, 4/week; so, coded 0.6 (corresponding to 4÷7=0.6/day), 1-3 times weekly would mean, on average, 2/week; so, coded 0.3; 1-3 times monthly would mean, on average, 2/month; so, coded 0.1; none would be zero (9). Each code was multiplied by the calcium content of that food item in gramme according to food composition tables (8) to get daily calcium intake obtained from each specified food. The total calcium intake was calculated by summation of calcium content of food items mentioned (9). Participants were subjected to a physical examination, including measurement of weight and height to evaluate the body mass index (BMI). Finally, measurement of BMD was done by a quantitative ultrasound densitometry method. This method is recommended for screening normal individuals for osteoporosis (10). It was done using GE Lunar Achilles Insight Bone Densitometer (a portable instrument measuring BMD at the foot made by GE Healthcare). Diagnostic criteria for osteoporosis (and low bone mass) have been proposed by an expert panel of the World Health Organization (11) (Figure).

Females diagnosed with osteopaenia or osteoporoses in this study received a health education session about the disease and were given a list of calcium-rich food items together with some advice regarding modifying their lifestyles, with special focus on sun exposure and exercise, they were also advised to consult their physicians.

### Analysis of data

Data collected were coded, analyzed, and interpreted using SPSS (version 16) provided by International Business Machines Corp. (IBM). Descriptive statistics were expressed as mean±SD or percentage. Differences between the groups were analyzed using the ANOVA test for continuous normally-skewed variables or Kruskal-Wallis test for non-parametric variables. The chi-square test, Fisher's exact test, or Monte Carlo test were used for categorical variables. Pearson's bivariate correlation test was done to reveal correlation between BMD and investigated factors. Linear regression analysis was used in estimating the association of variables of interest and T-score of BMD, taking into consideration eventual problems with multicollinearity. Multicollinearity is a condition in which independent variables that have a variance inflation factor (VIF) greater than 5 should be dropped out from the regression model as their presence in the model will result in significant variables to appear to be insignificant. VIF was calculated for each independent variable of interest, and all variables with values greater than 5 were dropped out of the regression model. A two-tailed p value of <0.05 was considered statistically significant.

### Ethical approval

The study protocol was approved by The IRB (Institutional Research Board) Committee at the CAMS.

**Figure.**
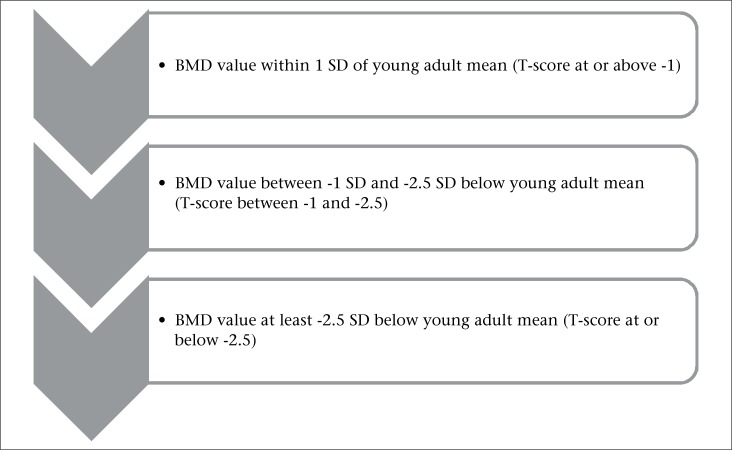
Diagnostic criteria for osteoporosis proposed by World Health Organization (11)

## RESULTS

### Characteristics of participants

Two hundred fifty-six females were selected, and 153 of them agreed to participate in the study (the response rate was 59.7%). Low response rate in the present study could be attributed to lack of interest towards osteoporosis. Besides, the time of study (May to June) could be a factor hindering their participation as this was the time for final exams at the college with accompanying workload.

Half of the participants were young adults aged 35 years or less and three-quarters of them was pre-menopausal women; 42.2% of the participants were regularly doing exercise. Less than half of the participants (46%) reported exposure to sun, with mean duration of exposure equalling only 5 minutes per day. The majority of participants (91.4%) had deficient daily calcium intake (less than 1,000 mg/day).

Table 1 demonstrates that 30.1% of the whole sample had osteopaenia, and most of them (63%) were pre-menopausal women while the overall prevalence of osteoporosis among the study participants was 6.5% that was equally distributed between pre- and post-menopausal women.

Comparison of pre- and post-menopausal women regarding fertility and co-morbidity revealed that post-menopausal women had significantly longer fertility period, more parity, and more co-morbidity than pre-menopausal women.

In Table 2, the distribution of osteopaenia and osteoporosis among the study sample, according to the presence of hypertension and diabetes mellitus, was demonstrated, and it revealed that having hypertension or diabetes was significantly correlated to osteopaenia and osteoporosis.

### Correlation between BMD and investigated risk factors of osteoporosis

In Table 3, Pearson's bivariate correlation test revealed significant correlation between low BMD and increasing age, number of co-morbidity, parity, fertility period, gynaecological age, menopausal duration, and presence of hypertension. It was also found that long fertility period was significantly correlated with number of offspring and higher risk of osteoporosis. The prevalence of osteoporosis among females with fertility period less than 20 years was 1.7% while, among those with fertility period more than 25 years, it was 9.7%. Neither lack of exercise nor limited sun exposure had a significant correlation with osteoporosis in this study. The present study also revealed a significant correlation between presence of co-morbidity and having osteoporosis. The highest prevalence of osteoporosis was found among hypertensive patients (20% of them were osteoporotic), followed by diabetic patients (16.7%).

**Table 1.** Distribution of osteopaenia and osteoporosis among pre- and post-menopausal women according to fertility and co-morbidity

Variable	Group						p value
	Normal		Osteopaenia		Osteoporosis		
	Pre-menopause	Post-menopause	Pre-menopause	Post-menopause	Pre-menopause	Post-menopause	
Fertility period	18.5±10.1	35.7±4.6	17±7.9	37.4±5.3	28.7±20	37±1.2	p<0.01**
Mean±SD	21.2±11.3		25.6±12.2		33.4±12.4		
Number of co-morbidity	1.5±0.6	2.1±0.9	1.4±0.6	2.1±0.8	2±1	2.8±0.4	p<0.05*
Mean±SD	0.5±0.8		0.6±0.9		1.5±0.9		
Parity	2.1±2.3	6.6±3.2	2.6±2.5	4.9±2.7	6±4.5	6.8±1.8	p<0.01**
Mean±SD	2.8±3		3.5±2.8		6.4±3		
Total	83 (85.6%)	14 (14.4%)	29 (63%)	17 (37%)	5 (50%)	5 (50%)	p<0.01**
	97 (63.4%)		46 (30.1%)		10 (6.5%)	

SD=Standard deviation;

*Statistically significant;

**Highly significant

**Table 2.** Distribution of osteopaenia and osteoporosis among the study sample according to the presence of hypertension and diabetes mellitus

Variable	Group			p value
	Normal	Osteopaenia	Osteoporosis	
Hypertension				
No	87 (68%)	36 (28.1%)	5 (3.9%)	p<0.01**
Yes	10 (40%)	10 (40%)	5 (20%)	
Diabetes mellitus				
No	79 (64.2%)	39 (31.7%)	5 (4.1%)	p<0.05*
Yes	10 (40%)	10 (40%)	5 (20%)		

*Statistically significant;

**Highly significant

Linear regression analysis was performed (Table 4) to determine which independent variables could predict the T-score of BMD. VIF was calculated for each independent variable in Table 3, and all variables with values greater than 5 were dropped. These were: age, number of co-morbidities, fertility period, gynaecological age, menopausal duration, and hypertension. So, only the parity (number of offspring) that had VIF=1.4 entered the regression model. It was found that parity maintained a significant association with T-score of BMD (p<0.05).

### Dietary habits

It was found that the higher the frequency of consuming cheese and eggs, the lower was the prevalence of osteoporosis. On analyzing the total calcium intake, the results revealed that the mean amount of calcium intake per day was lower among osteopaenic and osteoporotic patients than its mean among normal individuals (564.9±355.7, 425.8±306.7, and 567.3±331.3 respectively). However, these differences were not statistically significant (Table 5).

## DISCUSSION

In the present study, the prevalence of osteopaenia and osteoporosis among pre-menopausal women was 24.8% and 4.3% respectively, and it increased significantly with age which could be attributed to the decreased level of estrogen hormone at old age, with consequent negative effect on bone. The high prevalence of osteopaenia in pre-menopausal females indicates the ultimate importance of early intervention to prevent development of osteoporosis in later life; so, routine measurement of BMD every 1 year for all pre-menopausal females for early detection and treatment of osteopaenia seemed to be essential.

It was also found that the prevalence of osteopaenia was significantly higher than that of osteoporosis in all age-groups. For example, in women aged 45-60 years, the number of women with osteopaenia was six-fold higher than the number with osteoporosis, and it was 1.5-fold higher in the age-group of 60 years or more. Similar findings were reported in other studies. Kanis *et al*. (12) reported that the prevalence of osteoporosis in the Swedish females increased with age. Approximately 21% of women aged 50-84 years were classified as having osteoporosis compared to 50% women aged 80-84 years. He also reported that, in women aged 50–54 years, the number of individuals with low bone mass was six-fold higher than the number of those with osteoporosis. These results coincide with Demir *et al*. (13) who reported the prevalence of osteopaenia and osteoporosis among Turkish women aged 52.9±4.7 years to be 39.2% and 16.2% respectively. Regarding studies in Saudi Arabia, similar high prevalence of both osteopaenia and osteoporosis were reported, particularly among old-aged females. Desouki M (3) estimated the prevalence of osteopaenia and osteoporosis in post-menopausal Saudi women aged 50-80 years to be 31% and 40% respectively.

**Table 3.** Pearson's bivariate correlation between BMD and investigated risk factors

Variable	Pearson's correlation	Bone mass T-score
Age	Pearson's correlation	-0.318**
	Significant (2-tailed)	0.000
Hypertension	Pearson's correlation	-0.211**
	Significant (2-tailed)	0.009
Parity	Pearson's correlation	-0.196*
	Significant (2-tailed)	0.021
Gynaecological age	Pearson's correlation	-0.335**
	Significant (2-tailed)	0.000
Co-morbidity	Pearson's correlation	-0.173*
	Significant (2-tailed)	0.034
Fertility period	Pearson's correlation	-0.278**
	Significant (2-tailed)	0.001
Duration of menopause	Pearson's correlation	-0.351*
	Significant (2-tailed)	0.036

*Correlation is significant at 0.05 level (2-tailed);

**Correlation is significant at 0.01 level (2-tailed)

**Table 4.** Linear regression analysis of variable predicting T-score of BMD

Model	Unstandardized coefficients		Standardized coefficients		T-score	p	VIF
	B	SE	β				
Constant	-0.19	0.17		-1.1		
Parity	-8.7	0.04	-0.19	-2.3	<0.05	1.4

SE=Standard error; T=Corresponding t value; VIF=Variance inflation factor

**Table 5.** Correlation between calcium intake and BMD

Variable	Group			p value
	Normal	Osteopaenia	Osteoporosis	
Calcium intake				
Deficient calcium intake (<1,000 mg/day)	51 (60%)	28 (32.9%)	6 (7.1%)	p>0.05
Average intake (≥1,000 mg/day)	6 (75%)	2 (25%)	0 (0%)	
Mean±SD of calcium intake (mg/day)	567.3±331.3	564.9±355.7	425.8±306.7	p>0.05

SD=Standard deviation

The present study revealed significant correlation between low BMD and both increased parity and duration of fertility period in years. The correlation with increased parity could be attributed to two factors: first, the increased requirements of calcium during each pregnancy, which if not accompanied with adequate calcium intake, will lead to decreased BMD; second, increased number of offspring means longer duration of lactation and subsequent increased daily requirement of calcium. Besides possible lactation, amenorrhoea will also increase possibility of osteoporosis (14). On the other hand, the correlation of low BMD and long fertility period could be explained by the highly statistical correlation between fertility period and parity in such community that neglects the use of contraceptive methods due to religious, social and cultural issues (24% of Saudi women aged 15-49 years were using contraceptive methods) (15). It is worth mentioning that 21.6% of mothers enrolled in the study had 6 or more children. This study reported a strong correlation between osteoporosis and hypertension. Caudarella *et al*. (16) attributed such association to the high salt intake which leads to hypertension as well as increased calcium excretion in urine with subsequent negative calcium balance and greater bone loss. However, salt intake was not estimated among participants in the present study.

On studying dietary habits, it was found that the higher the frequency of consuming dairy products and eggs, the lower was the prevalence of osteoporosis; this coincided with results reported by Al Masri *et al*. (17) who studied the relationship between osteoporosis and eating habits during adolescence and early adulthood in a group of post-menopausal Jordanian women. They concluded that women who disliked milk were about 3 times more prone to developing osteoporosis compared to women who liked milk. Women who disliked yogurt during their adolescence and early adulthood were about 6 times more prone to osteoporosis compared to women who liked yogurt while women who disliked *Labneh* (concentrated yogurt) and white cheese were about 10 times more prone to developing osteoporosis compared to women who liked it. On analyzing the total calcium intake results, the present study revealed that the mean amount of calcium intake per day was lower among osteopaenic and osteoporotic patients than its mean among normal individuals; however, these differences were not statistically significant. This non-significance may be explained by the fact that the majority of participants (91.4%) had deficient daily calcium intake less than 1,000 mg/day, and only 8.6% of them consumed average daily calcium required. These results were documented by Fardellon (18) who conducted a cross-sectional survey in post-menopausal women and reported that the total mean daily dietary calcium intake in the study population was 754 mg/day; however, when the data were analyzed by class of calcium intake, 37.2% of the sample consumed less than 600 mg/day and only 20.1% over 1,000 mg/day. No difference in calcium intake was observed between 254 women reporting having osteoporosis and those who did not (p=0.43). Nguyen *et al.* (19) reported that dietary calcium intake was correlated modestly with BMD, although this relationship was apparent mainly in men and women with low body mass index less than 27 kg/m^2^.

It is worth mentioning that, despite living in such tropical area with sunrays available round the year, sun exposure was very limited among females. Wearing a veil cannot be considered a barrier hindering sun exposure because workplaces for women in Saudi Arabia are completely separated from that for men; so, almost all women do not wear the veil in workplace, and skin can be freely exposed to sunrays in the private garden in workplaces (In the college where the study was conducted, there was a medium-sized garden specified for women where they can get sun exposure daily); however, the very high temperature during most of the year that may reach 45 ºC or more is considered the biggest barrier hindering sun exposure.

### Limitations

This study acknowledges some limitations. This was a cross-sectional study which was suitable for evaluating the prevalence of osteoporosis. However, this design was not appropriate for assessing risk factors. Besides, low response rate and consequently small sample-size was another limitation facing reservation for generalization of its findings for the Saudi Arabian women in general.

### Conclusions

The present study documented that pre-menopausal females had high prevalence of osteopaenia (24.8%), which indicates the importance of early intervention to prevent development of osteoporosis at old age. There was a significant correlation between having osteoporosis and increasing age, fertility period, parity, menopausal duration, gynaecological age (time since menarche in years), and presence of co-morbidity, especially hypertension and diabetes mellitus. Parity was found to be an important predictor of T-score of BMD.

### Recommendations

- Health education campaigns for women, demonstrating the preventive measures of osteoporosis, need to be implemented.
- The ultimate importance of sun exposure and exercise should be explained to women during health education campaigns.
- Health education programmes concerning reproductive health for females should be strengthened to raise their awareness regarding the association between parity and BMD.
- Special emphasis should be given on adequate daily calcium intake, its dietary sources, and the use of calcium supplement during pregnancy and lactation.

## ACKNOWLEDGEMENTS

The authors acknowledge the help of Dr. Mona El Shafei who planned and facilitated using GE Lunar Achilles Insight Bone Densitometer for measuring BMD. The authors also thank all the subjects who participated in the study.

## References

[1] Looker AC, Melton LJ, Harris TB, Borrud LG, Shepherd JA (2010). Prevalence and trends in low femur bone density among older US adults: NHANES 2005-2006 compared with NHANES III. J Bone Miner Res.

[2] Mahussain S, Badr H, Al-Zaabi K, Mohammad M, Alnafisi N (2006). Bone mineral density in healthy Kuwaiti women. Arch Osteoporos.

[3] El-Desouki MI (2003). Osteoporosis in postmenopausal Saudi women using dual x-ray bone densitometry. Saudi Med J.

[4] Handa R, Kalla AA, Maalouf G (2008). Osteoporosis in developing countries. Best Pract Res Clin Rheumatol.

[5] Bridgeman MB, Pathak R (2011). Denosumab for the reduction of bone loss in postmenopausal osteoporosis: a review. Clin Ther.

[6] Kleerekoper M, Al-Khayer F (2004). Osteoporosis, overview. Encyclopedia Endocr Dis.

[7] Keramat A, Patwardhan B, Larijani B, Chopra A, Mithal A, Chakravarty D (2008). The assessment of osteoporosis risk factors in Iranian women compared with Indian women. BMC Musculoskelet Disord.

[8] Rolfes S, Pinna K, Whitney E (2009). Understanding normal and clinical nutrition.

[9] Willett WC (1998). Nutritional epidemiology.

[10] Mondry A, Hetzel GR, Willers R, Feldkamp J, Grabensee B (2001). Quantitative heel ultrasound in assessment of bone structure in renal transplant recipients. Am J Kidney Dis.

[11] World Health Organization (1998). Guidelines for preclinical evaluation and clinical trials in osteoporosis.

[12] Kanis JA, Johnell O, Oden A, Jonsson B, De Laet C, Dawson A (2000). Risk of hip fracture according to the World Health Organization criteria for osteopenia and osteoporosis. Bone.

[13] Demir B, Haberal A, Geyik P, Baskan B, Ozturkoglu E, Karacay O (2008). Identification of the risk factors for osteoporosis among postmenopausal women. Maturitas.

[14] Cumming RG, Klineberg RJ (1993). Breastfeeding and other reproductive factors and the risk of hip fractures in elderly women. Int J Epidemiol.

[15] United Nations (2011). World contraceptive use 2010.

[16] Caudarella R, Vescini F, Rizzoli E, Francucci CM (2009). Salt intake, hypertension, and osteoporosis. J Endocrinol Invest.

[17] Al-Masri B The relationship between osteoporosis and eating habits during adolescence and early adulthood. Paper presented on Second Pan-Arab Osteoporosis Congress. Third International Congress of the Egyptian Osteoporosis Prevention Society, Sharm El Sheikh, 22-25 October 2002:19-25.

[18] Fardellone P, Cotté F-E, Roux C, Lespessailles E, Mercier F, Gaudin A-F (2010). Calcium intake and the risk of osteoporosis and fractures in French women. Joint Bone Spine.

[19] Nguyen TV, Center JR, Eisman JA (2000). Osteoporosis in elderly men and women: effects of dietary calcium, physical activity, and body mass index. J Bone Miner Res.

